# Development and Validation of a Two-Step Predictive Risk Stratification Model for Coronavirus Disease 2019 In-hospital Mortality: A Multicenter Retrospective Cohort Study

**DOI:** 10.3389/fmed.2022.827261

**Published:** 2022-04-07

**Authors:** Yang Li, Yanlei Kong, Mark H. Ebell, Leonardo Martinez, Xinyan Cai, Robert P. Lennon, Derjung M. Tarn, Arch G. Mainous, Aleksandra E. Zgierska, Bruce Barrett, Wen-Jan Tuan, Kevin Maloy, Munish Goyal, Alex H. Krist, Tamas S. Gal, Meng-Hsuan Sung, Changwei Li, Yier Jin, Ye Shen

**Affiliations:** ^1^Center for Applied Statistics and School of Statistics, Renmin University of China, Beijing, China; ^2^RSS and China-Re Life Joint Lab on Public Health and Risk Management, Renmin University of China, Beijing, China; ^3^Department of Epidemiology and Biostatistics, College of Public Health, University of Georgia, Athens, GA, United States; ^4^Department of Epidemiology, School of Public Health, Boston University, Boston, MA, United States; ^5^Department of Family and Community Medicine, Penn State College of Medicine, Hershey, PA, United States; ^6^Department of Family Medicine, David Geffen School of Medicine at UCLA, University of California, Los Angeles, Los Angeles, CA, United States; ^7^Department of Health Services Research, Management and Policy, University of Florida, Gainesville, FL, United States; ^8^Departments of Family and Community Medicine, Public Health Sciences, and Anesthesiology and Perioperative Medicine, Penn State College of Medicine, Hershey, PA, United States; ^9^Department of Family Medicine and Community Health, University of Wisconsin, Madison, WI, United States; ^10^Department of Emergency Medicine, MedStar Washington Hospital Center, Washington, DC, United States; ^11^Department of Family Medicine and Population Health, Virginia Commonwealth University, Richmond, VA, United States; ^12^Department of Biostatistics, Virginia Commonwealth University, Richmond, VA, United States; ^13^Department of Epidemiology, Tulane University School of Public Health and Tropical Medicine, New Orleans, LA, United States; ^14^Department of Electrical and Computer Engineering, University of Florida, Gainesville, FL, United States

**Keywords:** prognostic score, two-step, time-and cost-saving tool, COVID-19, multicenter cohort study

## Abstract

**Objectives:**

An accurate prognostic score to predict mortality for adults with COVID-19 infection is needed to understand who would benefit most from hospitalizations and more intensive support and care. We aimed to develop and validate a two-step score system for patient triage, and to identify patients at a relatively low level of mortality risk using easy-to-collect individual information.

**Design:**

Multicenter retrospective observational cohort study.

**Setting:**

Four health centers from Virginia Commonwealth University, Georgetown University, the University of Florida, and the University of California, Los Angeles.

**Patients:**

Coronavirus Disease 2019-confirmed and hospitalized adult patients.

**Measurements and Main Results:**

We included 1,673 participants from Virginia Commonwealth University (VCU) as the derivation cohort. Risk factors for in-hospital death were identified using a multivariable logistic model with variable selection procedures after repeated missing data imputation. A two-step risk score was developed to identify patients at lower, moderate, and higher mortality risk. The first step selected increasing age, more than one pre-existing comorbidities, heart rate >100 beats/min, respiratory rate ≥30 breaths/min, and SpO_2_ <93% into the predictive model. Besides age and SpO_2_, the second step used blood urea nitrogen, absolute neutrophil count, C-reactive protein, platelet count, and neutrophil-to-lymphocyte ratio as predictors. C-statistics reflected very good discrimination with internal validation at VCU (0.83, 95% CI 0.79–0.88) and external validation at the other three health systems (range, 0.79–0.85). A one-step model was also derived for comparison. Overall, the two-step risk score had better performance than the one-step score.

**Conclusions:**

The two-step scoring system used widely available, point-of-care data for triage of COVID-19 patients and is a potentially time- and cost-saving tool in practice.

## Introduction

Coronavirus disease 2019 (COVID-19), the infectious disease resulting from severe acute respiratory syndrome coronavirus 2 (SARS-CoV-2), has led to morbidity and mortality in millions of people (1). A simple, reliable, point-of-care risk score to predict mortality could help clinicians triage patients and appropriately allocate resources. This is particularly important as health systems face shortages of hospital intensive care unit (ICU) beds that can lead to worse clinical outcomes (2).

Various prognosis scores have been proposed to achieve this goal (3–6). Several models have used varying combinations of demographic variables, laboratory tests, or imaging (7–10). Tools that provide accurate, low-cost risk estimates are needed, as estimates requiring extensive testing or imaging increase the burden on healthcare systems already operating at capacity. Prognostic tools based on data combined from different regions or countries (11–13) are problematic, as they ignore heterogeneity between populations that may increase the risk of bias (3). While the extent of this risk across all regions is not well elucidated, it has been demonstrated in one regional comparison by the ISARIC 4C Deterioration model (13).

We developed an easy-to-use, practical clinical prediction rule for mortality in patients with COVID-19, building on a conceptual framework of a two-step triage (14). With the proposed two-step procedure, early identification of lower- and higher- risk groups and accurate patient triage are possible while conserving limited resources. We validated our model on distinct external cohorts across various populations to fully characterize heterogeneity across settings and clinical presentation.

## Materials and Methods

### Derivation Cohort and Validation Cohorts

Four universities with inpatient health centers including Virginia Commonwealth University (VCU), Georgetown University (GU), University of Florida (UFL), and University of California, Los Angeles (UCLA) participated in the study. Data were retrospectively extracted from electronic health records (EHRs) of each health system. The cohort from VCU, with the longest patient enrollment period (from March 2020 to June 2021) among centers, was used as the derivation cohort and the remaining three university health system cohorts were used for validation to assess model performance in heterogeneous populations.

### Study Participants and Data Collection

Participants included from each center were hospitalized adults (18 years old and above) with a positive polymerase chain reaction (PCR) test for SARS-CoV-2 and a determined disposition (discharged or deceased) at the time of data extraction. The diagnosis of SARS-CoV-2 infection was based on World Health Organization interim guidance (15). The outcome of interest was in-hospital mortality, documented in each patient's EHR-based hospital disposition.

Data collection of the four cohorts all started in March, 2020. The derivation cohort VCU possessed the latest patient information by June, 2021. GU included data collection from March to August, 2020. Data of UFL was last updated by December, 2020, while the UCLA cohort enrolled patients until May, 2021. Demographic, clinical, and laboratory variables were extracted from the EHRs following the standardized approach to each variable definition (6). Those variables were divided into routinely available and laboratory available categories. Routinely available predictors included age, gender, vital signs, physical examination results such as heights and weight that generate body mass indexes (BMIs), and number of comorbidities. Comorbidities were defined using Clinical Classifications Software categories for diabetes mellitus (CCS 49), cardiovascular disease (CVD, CCS 101), asthma (CCS 128), and chronic obstructive pulmonary disease (COPD, CCS 127) (16), then these comorbidities were combined to create a count variable. Laboratory available predictors were commonly used laboratory test measurements (white blood cell count, neutrophil count, lymphocyte count, creatinine, platelets, blood urea nitrogen, lactate dehydrogenase, aspartate aminotransferase, alanine aminotransferase, C-reactive protein, and troponin-I). Only the first measured predictor variables available within 24 h of admission date/time were included.

### Model Development

We developed a two-step risk score using an approach similar to that used by Fine and colleagues to develop the Pneumonia Severity Index (14). The study followed the Transparent Reporting of a multivariable prediction model for Individual Prognosis or Diagnosis (TRIPOD) principles (17). The first step was designed for rapid identification of lower- and higher- risk groups; the second step was for classification of the remaining patients using additional and more-difficult-to-obtain predictor variables.

Before model development, numerical variables were categorized according to their clinical normal ranges (18, 19). The neutrophil-to-lymphocyte ratio (NLR) was computed using extracted values (20, 21), and its dichotomous cutoff was derived from the max Youden index (22) of a univariable binary logistic model. Only categorical variables were used for model fitting. The multiple imputation (MI) method was applied for missing values of candidate predictor variables. Under the assumption of missing at random, a chained equations approach (23) carried out five imputations. We used Rubin's rules (24) to combine the model parameter estimates across the imputed datasets.

**Figure 1:**
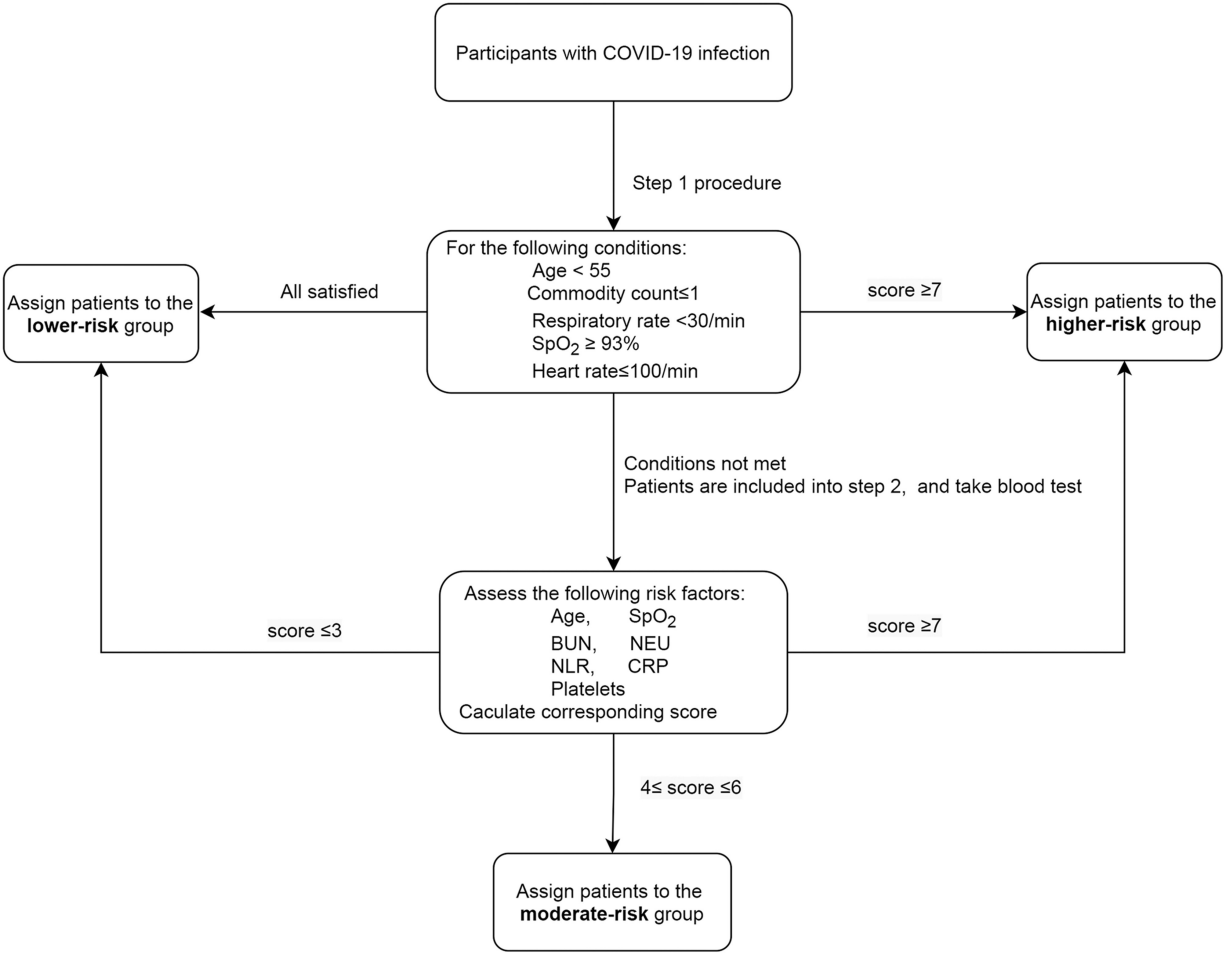
Two-step algorithm for assessing mortality risk from SARS-CoV-2 infection.

The developed algorithm involves two steps as shown in the flowchart in Figure 1. In the first step, only routinely available variables like demographics and vital signs were included as candidate predictors. Step 1 applied the MI-stepwise method (25) with a likelihood-ratio test statistic to select risk factors. We repeated the variable selection procedure 100 times and included those that were selected over 50 times. Then, a multivariate binary logistic model was employed with relaxed inclusion criteria (*P* ≤ 0.1) to include more risk factors. After parameter estimation, each beta-coefficient was divided by the smallest one and subsequently rounded to the nearest integer to create a simple point score (18). The risk score was calculated additively. Patients with the lowest observed-cumulative mortality in Step 1 were classified into the lower-risk group, and those with observed-cumulative mortality >30% were classified into the higher-risk group. The corresponding observed mortality of the two groups was then used as the lower- and higher-risk cutoffs in the next step (11, 14). Patients who were not assigned to either the lower- or higher- risk group in the first step then participated in the second step. Both routinely available and laboratory available variables were taken into consideration for the second stage model. Step 2 conducted a similar procedure to develop its risk score as for Step 1, categorizing remaining individuals into lower-, moderate-, and higher-risk groups based on the corresponding observed cumulative mortality.

### Model Validation

Complete datasets from each of the four health systems were used to evaluate the performance of the proposed risk score. The cohort from VCU was used for internal validation, while complete cases from the remaining health centers were used separately for external validation. The number of patients in each risk group and the corresponding mortality rate for each risk group were calculated for each health system cohort. Cochran-Armitage tests (26) were used to test for trends in mortality from an increasing number of points and classification categories. We also employed the Gaussian mixture model (GMM) (27) at the second step to assess the rationality of clustering and the consistency of risk group separation with the first step. Overall discrimination ability was assessed by C-statistics (28) with a corresponding 95% confidence interval. Calibration curves (29) and the Hosmer-Lemeshow test (30) were used to evaluate how well the predicted mortality matched the observed mortality. Sensitivity analysis was conducted using complete case data to assess the MAR missing assumption and to evaluate the goodness of MI-stepwise two-step method.

### Comparison With Direct Risk Stratification

Traditional mortality predictive scores are often derived from direct logistic models to create single one-step risk scores (3–6). We used all risk factors available and employed the one-step model-fitting method on the derivation cohort (*P* ≤ 0.05). After calculation of mortality scores, patients were classified into three groups according to the same observed cumulative mortality cutoffs of the two-step method. Model validation was also conducted on the complete cases for each cohort.

To compare the performance of the two methods, we assessed discrimination and calibration using C-statistics (28) and Brier scores (31), respectively. For those patients whose probability of death could not be evaluated due to missing variables needed for prediction, we also conducted MI-imputation using demographic variables and vital signs for mortality estimation. Decision curve analysis (32) was subsequently employed to compare the clinical utility of the two models at different risk thresholds. Briefly, by assuming a threshold probability for the higher mortality risk, we can derive the net benefit by weighing the benefit of the true-positive and the cost of the false-positive prediction. The net benefit curve obtained from different threshold probabilities reflects the clinical utility of a model. Two extreme strategies in which either all or none of the patients were classified to the higher-risk group served as reference points.

### Ethics Approval

The overall study protocol was approved by the Institutional Review Board at the University of Georgia under approval number: PROJECT00002208.

## Results

### Patient Demographic and Clinical Characteristics

The derivation cohort included 1,673 adults with PCR confirmed COVID-19, with 180 (10.8%) deaths. GU, UFL and UCLA had 558, 1,815 and 1,570 individuals, with 93 (16.7%), 269 (14.8%), 184 (11.7%) deaths, respectively. We summarized continuous variables as medians with interquartile ranges and categorical variables using proportions (Supplementary Table S1). The missing proportion of collected variables in the VCU cohort is shown in Supplementary Table S2.

### Development of Predictive Risk Stratification of Two-Step Methods

In step 1, 63 (3.77%) individuals of the derivation cohort (VCU) had missing information for routinely available variables. The repeated MI-stepwise variable selection procedure identified age above 55, more than one pre-existing comorbidities, heart rate >100 beats/min, respiratory rate >30 breaths/min, and SpO_2_ <93% as the most important predictors for mortality (Suppmentary Tables S3, S4). Individuals who scored zero, without any of these risk factors, were classified into a lower-risk group. While patients with score ≥7 were considered as having relatively high risk of death, admitted into the higher-risk group (Figure 1). The corresponding observed cumulative mortality cutoffs was then used as the corresponding thresholds in the second step. In step 2, 1,155 patients from the remaining patients (*n* = 1,220) had missing information. Repeated MI-stepwise procedure showed that besides age and SpO_2_, laboratory variables including blood urea nitrogen (BUN), neutrophils absolute count, C-reactive protein (CRP), platelets count and NLR also had significant influences on the mortality rate (Supplementary Tables S5, S6). The final risk score is shown in Table 1.

**Table 1:** The proposed two-step risk score for coronavirus disease 2019 mortality.

**Predictors of step 1**	**Points**	**Risk group**	**Points**
Age (years)		Lower risk	0
<55	0	Higher risk	≥ 7
55–64	2	Go to Step 2	1–6
65–74	3		
≥75	5		
Respiratory rate ≥30	2		
SpO_2_ <93%	2		
Commodity count ≥2	1		
Heart rate >100	1		
**Maximum**	11		
**Predictors of step 2**	**Points**	**Risk group**	**Points**
Age (years)		Lower risk	≤ 3
<55	0	Moderate risk	4–6
55–64	1	Higher risk	≥7
65–74	2		
≥75	3		
SpO_2_ <93%	2		
BUN > 20 mg/dl	2		
NLR > 3.7	2		
NEU >6.3	2		
Platelets ≥350	2		
CRP >10	1		
**Maximum**	14		

### Independent Validation of Two-Step Rule

#### Internal Validation

**Figure 2:**
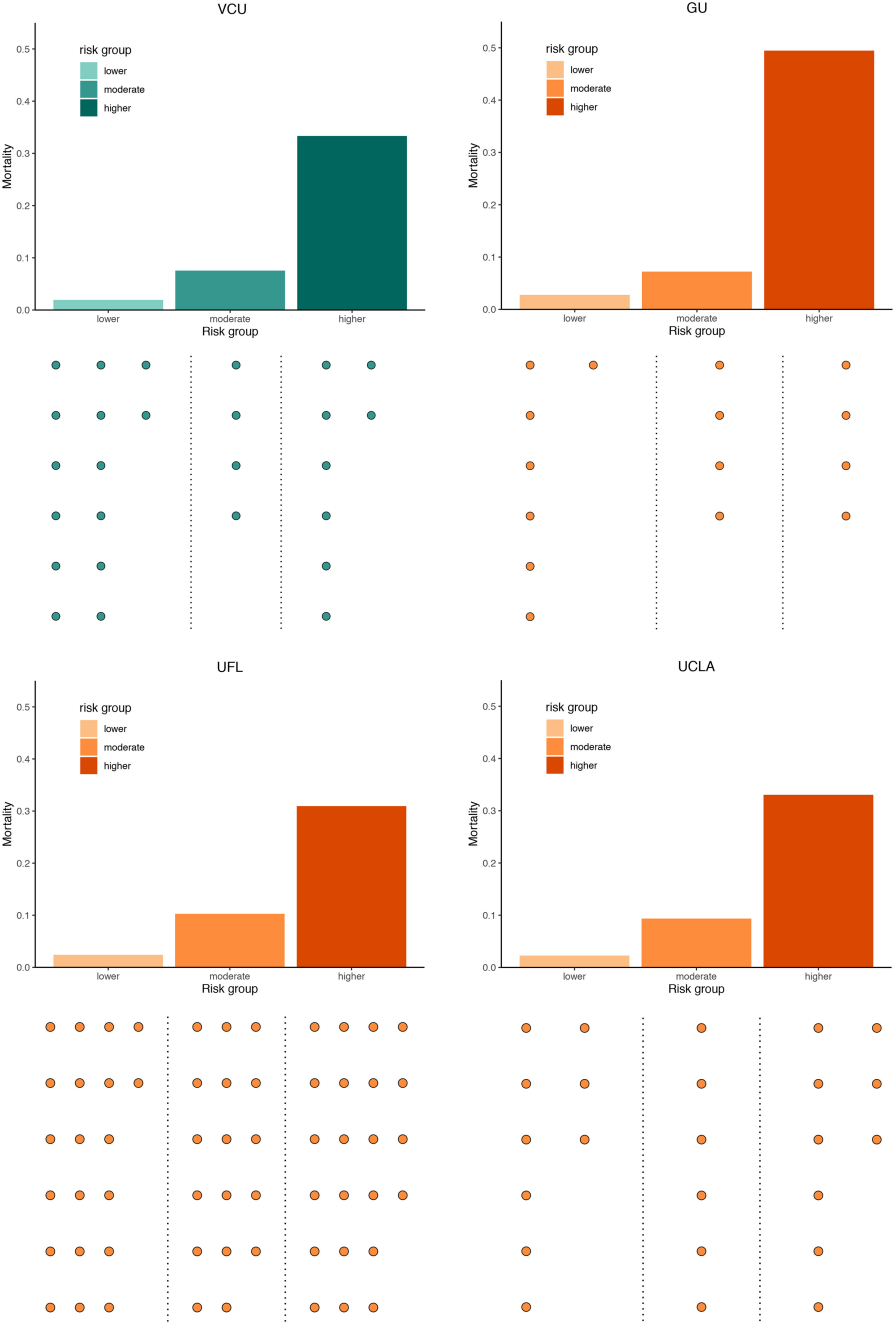
Risk Stratification Among Derivation and Validation Cohorts. Bar plots represented mortality risk. A dot below each main plot represented five people within each corresponding group, and the number of dots suggests the approximate sample size in each group.

**Figure 3:**
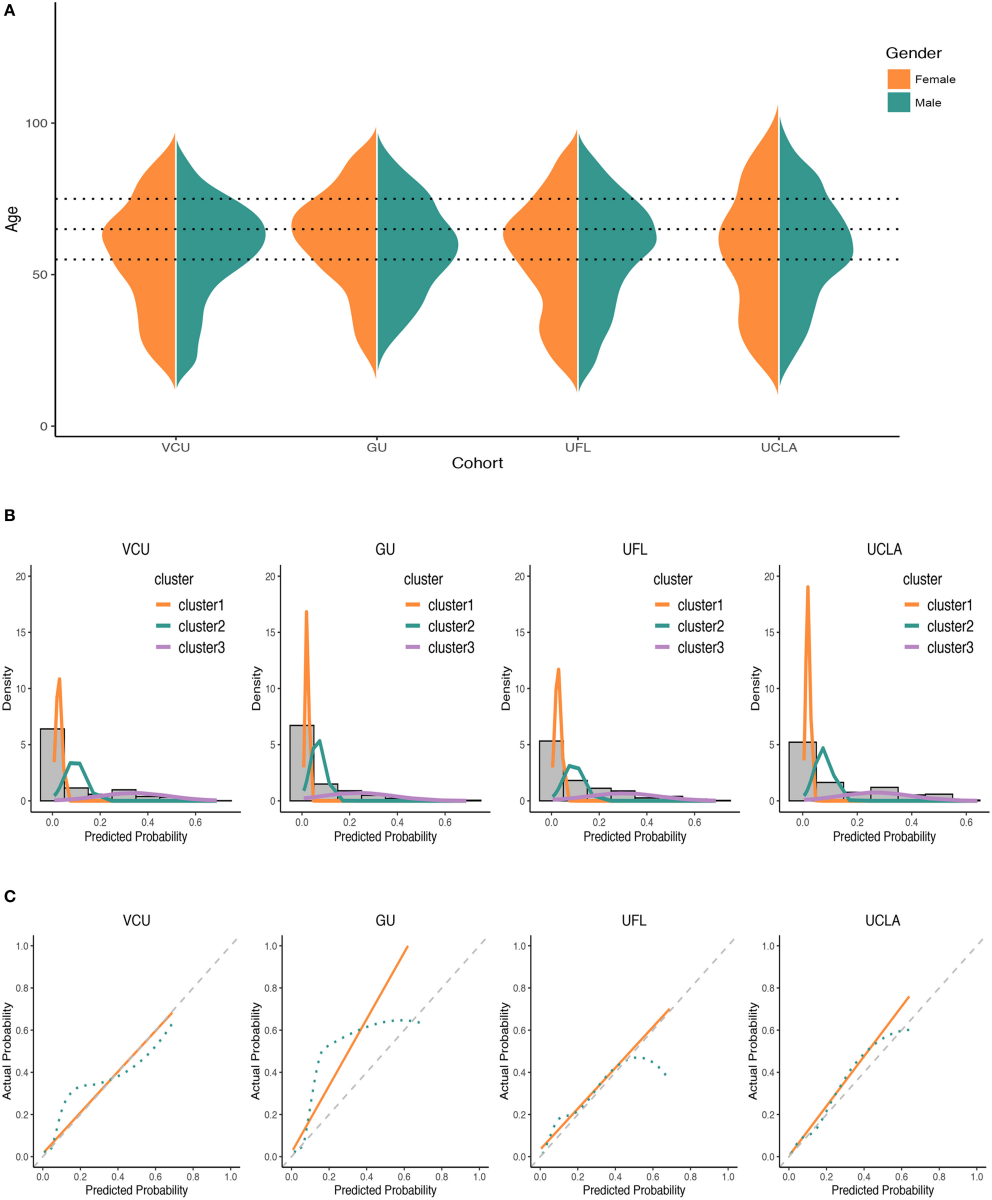
Distribution of demographic variables, discrimination, and calibration ability of the two-step method in the derivation cohort. **(A)** Distributions of demographic variables. **(B)** GMM distributions among different cohorts. **(C)** Calibration curves for two-step method using logistic calibration and locally weighted scatterplot smoothing (lowess). The dot lines in **(A)** were age cutoffs. The first cluster line plot of VCU in **(B)** was truncated for convenient comparison with other cohorts. The actual peak of this line was at around 95.

When the derived predictive risk stratification was applied, mortality rates in step 1 were 2.0% and 30.1% in the lower- and higher-risk groups, respectively. Patients assigned to the lower-, moderate-, and higher-risk groups for step 2 had an observed mortality rate of 1.8, 7.6 and 35.5%, consistent with results from step 1. We merged patients of the two steps together to evaluate the overall death rates of each group, and the corresponding mortality rates were 1.9, 7.6 and 33.3% (Table 2; Figure 2), resulting in good separation among the risk groups. Mortality risk had an increasing trend (*P*_trend_ < 0.001) among groups. GMM on the score-based predicted probability of the second step indicated significantly different risk profiles between the three groups (Figure 3B). The C-statistic was 0.83 (95% CI, 0.79–0.88) with good overall discrimination ability. The calibration curve (Figure 3C) suggested that predicted and observed mortality matched well (Hosmer–Lemeshow test, *P* = 0.995).

**Table 2:** Validation of the two-step coronavirus disease 2019 risk score in 4 populations #.

**Risk group**	**Internal validation**	**External validation cohorts**			
	**VCU**	**GU**	**UFL**	**UCLA**	**All External Validation**
Lower	1.9% (7/362)	2.8% (5/180)	2.4% (12/499)	2.3% (5/220)	2.5% (22/899)
Moderate	7.6% (8/106)	7.2% (7/97)	10.3% (44/428)	9.4% (13/139)	9.6% (64/664)
Higher	33.3% (68/204)	49.5% (45/91)	31.0% (173/559)	33.1% (77/233)	33.4% (295/883)
AUROCC^*^	0.832	0.854	0.793	0.829	0.825

* *AUROCC, Area under the receiver operating characteristic (ROC) curve*.

# *Numbers in parentheses were listed as deaths/total*.

#### GU as External Validation

The external validation in the GU cohort showed an overall good stratification. The mortality of the lower-risk group identified in the first step was 3.2%, while the higher-risk group had death count of 13 in total 19 cases (death rate: 68.4%). Risk probabilities classified by the second step in lower-, moderate- and higher-risk groups were 1.2, 7.2, and 44.4%, respectively. Overall risk stratification demonstrated a similar trend (Table 2; Figure 2). An increasing trend was suggested by the Cochran-Armitage test (*P*_trend_ < 0.001). GMM curves (Figure 3B) also identified the existence of 3 groups of the remaining people. The C-statistic was 0.85 (95% CI, 0.80–0.91). Calibration curve showed a deviation (Figure 3C), yet the *P*-value of the Hosmer-Lemeshow test was 0.080.

#### UFL as External Validation

278 people were identified in the lower-risk group at the first step and 7 of them died (2.5%), while 149 individuals in the higher-risk group with 63 death cases (42.3%). Overall corresponding mortality rates were 2.3, 10.3, and 26.8% of the lower-, moderate-, and higher-risk groups (*P*_trend_ < 0.001) (Table 2; Figure 2). The GMM curves (Figure 3B) also supported three risk clusters among step 2-remaining patients. The validation in the UFL cohort showed slightly less differentiable observed risks among different groups, with a C-statistic at 0.79 (95%CI, 0.76–0.82). Calibration curve displayed satisfactory calibration. Corresponding *P*-value of the Hosmer-Lemeshow test was 0.197.

#### UCLA as External Validation

Mortality rates derived from the first step of the UCLA validation for the lower- and higher-risk groups were 3.5 and 46.8%. Observed probabilities of the lower-, moderate-, and higher-risk groups in step 2 were 0.9, 9.4, and 21.0%, respectively (Table 2; Figure 2). Stratification of overall risk was consistent with other cohorts (lower: 2.3%, moderate: 9.4%, higher: 33.1%). The UCLA cohort also presented an increasing trend of risk (*P*_trend_ < 0.001) across risk groups. GMM curve (Figure 3B) implied a 3-level risk stratification. C-statistic was 0.83 (95%CI, 0.79–0.87). *P*-value of the Hosmer-Lemeshow test was 0.968.

### Sensitivity Analysis Using Complete Case Analysis

Our sensitivity analysis showed that both steps using complete cases selected similar variables to those selected by MI-stepwise procedure (Supplementary Tables S7, S8). Except for age, scores assigned to each level of selected risk factors remained the same as those assigned using the multiple imputation (MI) based two-step method. Besides, the two-step method using MI had better discrimination (Supplementary Table S9) and calibration (Supplementary Figure S1) abilities than the approach using only complete cases.

### Comparison With the Direct Method

The one-step direct method identified age, SpO_2_, blood urea nitrogen and C-reactive protein (CRP), white blood cell count, platelets count, and NLR as predictors. The score in each reference group was assigned to 0. Three older age groups (score: 1, 2, 2), SpO_2_ below 93% (score: 1), above normal levels of laboratory variables including BUN (score: 2), CRP (score: 1), platelets count (score: 2), white blood cell count (score: 1), as well as NLR (score: 2) were associated with elevated death risk. A total score was obtained by summing all points each subject received, after which patients were directly classified into lower- (below 2 points), moderate- (3–5 points), and higher-risk (6 and above points) groups. More details are provided in Supplementary Tables S10–S12.

**Figure 4:**
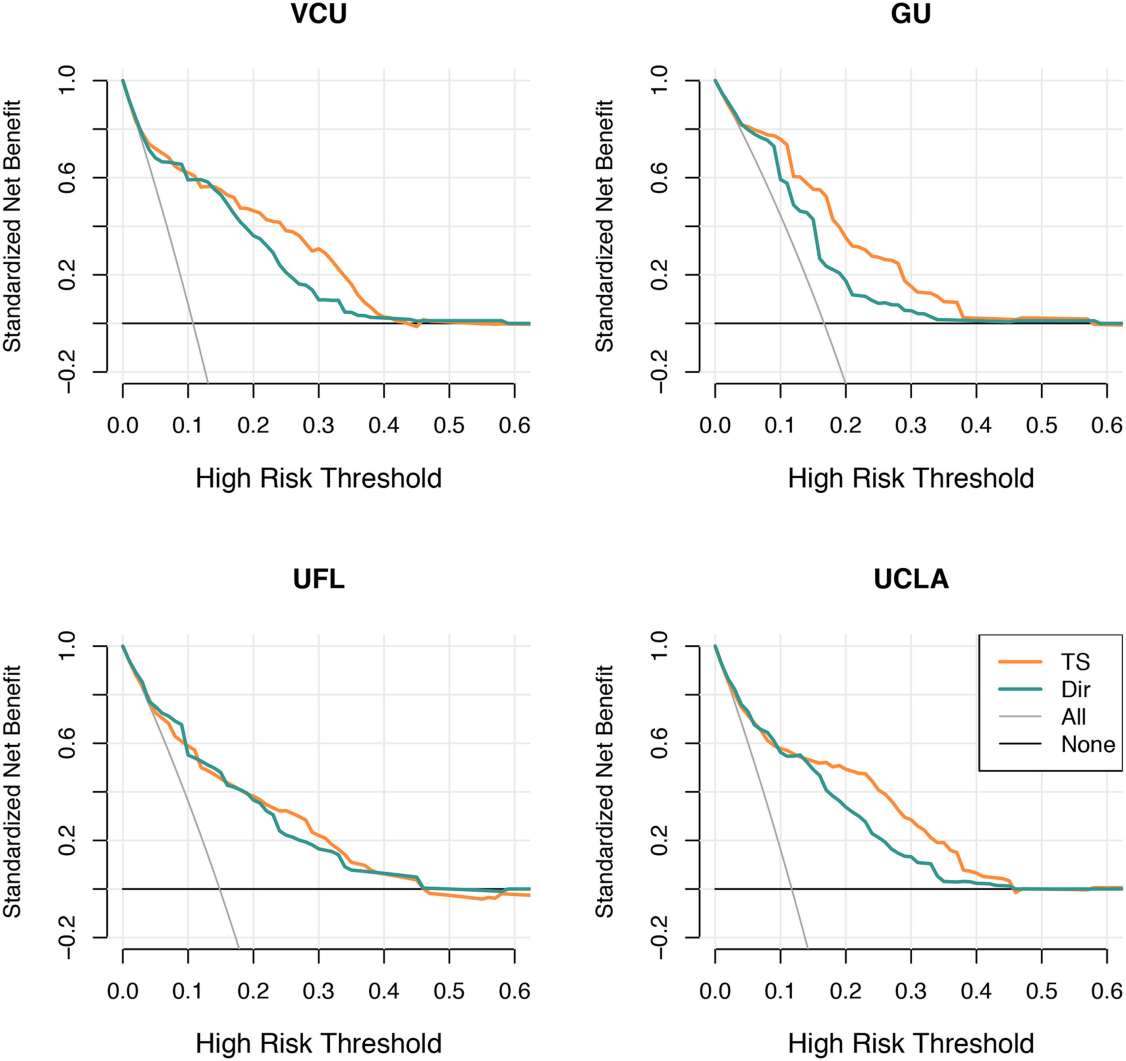
Decision curve analysis plots for a comparison of net benefits at different risk thresholds between the two-step (orange line) method and the direct one-step (green line) method.

The two-step method (TS) had better C-statistics and brier scores than the one-step direct method (OS) (Table 3). Net benefit curves (Figure 4) were generated based on thresholds of score-derived probabilities to evaluate clinical utilities. The higher net benefits observed from the two-step method in VCU, GU and UCLA suggested that it benefits more people at the population level in these regions. In the UFL cohort, the two methods resulted in comparable net benefits. Compared with the one-step method, the two-step risk score classified additional 331, 77, 165, and 136 subjects into the lower- or higher-risk groups in VCU, GU, UFL, and UCLA cohorts, respectively. Over half of the individuals triaged in the first step of the two-step method would be uncategorized by the one-step method due to missing lab testing predictors (Supplementary Table S13).

**Table 3:** C-statistics and brier score comparison between the two-step method (TS) and direct one-step (OS) method.

		**VCU**	**GU**	**UFL**	**UCLA**
**C-statistics** **(95% CI)**	TS	0.83 (0.79,0.88)	0.85 (0.80,0.91)	0.79 (0.76,0.82)	0.83 (0.79,0.87)
	OS	0.82 (0.77,0.88)	0.81 (0.74,0.87)	0.79 (0.76,0.82)	0.79 (0.74,0.84)
**Brier Score**	TS	0.09	0.11	0.11	0.11
	OS	0.12	0.12	0.12	0.11

## Discussion

SARS-CoV-2 has resulted in a growing number of deaths and a shortage of medical resources. Improved clinical prediction and decision support tools, feasible for implementation “at the bedside,” are urgently needed. Various scoring methods have been proposed (3–6) to achieve this goal with additional testing including laboratory exams, CT imaging, amongst others, leading to increased time and costs for patients and hospitals. We developed a simple, quick, and practical two-step predictive mortality score system for adult COVID-19 patient triage. The first step uses only routinely available characteristics that are easily collected to identify individuals with lower and higher mortality risk. The second step assesses the remaining patients comprehensively using both routinely available and laboratory data. The score system was validated in cohorts from multiple regions in the United States and achieved overall satisfactory prediction. Those validation cohorts were also collected over different time courses. The relatively stable performance adds strength to the generalizability and future applications of the study findings.

In comparison, the two-step model had better overall discrimination and calibration than the direct one-step method (Table 3; Supplementary Figure S2). The primary strength of the two-step approach is the time and money saved by appropriately stratifying patients using only easy-to-collect and routinely available variables, e.g., no imaging information needed, and no lab tests needed unless you get to the second step. The first step in the two-step method also ensures a higher coverage of all SARS-CoV-2 infected patients, which eventually would benefit a larger population. Overall, more than half of the individuals identified as lower or higher risk in the first step of the two-step method would otherwise be left uncategorized by the one-step method due to missing laboratory testing predictors. The number of lower- and higher-risk individuals identified by the two-step method in the first step can be regarded as the “benefit” of using this two-step procedure. Rapid, accurate triage may improve timely decision making, particularly for those patients missed by the one-step method.

To assess the performance of the two-step method in heterogeneous populations, we validated the score system using multiple external cohorts. As expected, model performance varied. Across derivation and validation cohorts, UFL performed worse than other cohorts, possibly because of geographic variability and a surprising increase of mortality in that cohort in late 2020. Age and gender differences could have contributed to the observed heterogeneity, as the four cohorts showed disparities in age distributions stratified by gender (Figure 3A). Racial diversity and its associated social economic status, underlying health conditions, healthcare access, and care-seeking behavior may also be important factors influencing mortality (33–35). As a surrogate for racial heterogeneity across the cohorts, we obtained state-level racial diversity information for each site (36). The derivation cohort from Virginia had comparable racial distribution with Delaware (where GU is located). By comparison, Florida and California had distinct racial profiles potentially explaining the suboptimal validation performance from the UFL and UCLA cohorts. Overall, the results suggest that the two-step model is suitable for each of these regions, but also identified regional heterogeneity that should be further explored for model refinement. Prospective, regional studies are needed to assess heterogeneity bias more precisely.

There are several limitations to this study. Coronavirus mutations may alter the course of the disease, and the proposed two-step method needs further validation in patients infected with emerging SARS-CoV-2 variants. Variant information was not available in our datasets, though based on the timeframe of our data collection the majority of our enrolled patients were likely infected with the wild-type. Further validation of the proposed approach and possible development of new triage scores on cohorts with new and existing SARS-CoV-2 variants are warranted. Data on vaccination status was also unavailable in our cohorts, which precludes an assessment of the effect of vaccination. Based on our data collection period and the current knowledge that the vaccinated population is at a significantly reduced risk of hospitalization, we consider our study findings mainly apply to the unvaccinated population. In addition, only the first measured predictor variables available within 24 h of admission date/time were included in developing the prediction model. It is unknown at what point in the disease's course a patient was admitted. Early or late enrollment in the cohort could result in false negative or false positive results in the higher-risk group. However, subjects included in our study were hospitalized patients who likely had been infected beyond the incubation period before admitted to the hospitals. They were all sick enough to present symptoms to be initially admitted for inpatient care. As such, our primary purpose is to assist in initial triage when these patients present. We suspect including days from initial symptom onset as a potential predictor in the model may further improve the prediction accuracy and reduce the bias caused by false negative or false positive predictions. Unfortunately, our working datasets did not collect this information.

## Conclusions

The proposed two-step score system for COVID-19-related in-hospital mortality among adults is time and cost-saving and may decrease health care burden in settings with high COVID-19 infection rates.

## Data Availability Statement

The data analyzed in this study is subject to the following licenses/restrictions: Medical records data. Requests to access these datasets should be directed to ebell@uga.edu.

## Ethics Statement

The studies involving human participants were reviewed and approved by the University of Georgia IRB. Written informed consent for participation was not required for this study in accordance with the national legislation and the institutional requirements.

## Author Contributions

YL, YK, and YS contributed to the conception and design of the study. RL, DT, AM, AZ, BB, W-JT, KM, MG, AK, and TG organized databases from each study site. ME, XC, and YK organized the combined database. YL, YK, and YS performed the statistical analysis. YL, YK, ME, LM, and YS wrote the first draft of the manuscript. M-HS, CL, and YJ provided technical supports to the manuscript. All authors contributed to manuscript revision, read, and approved the submitted version.

## Funding

YL is supported by Platform of Public Health & Disease Control and Prevention, Major Innovation & Planning Interdisciplinary Platform for the Double-First Class Initiative, Renmin University of China.

## Conflict of Interest

The authors declare that the research was conducted in the absence of any commercial or financial relationships that could be construed as a potential conflictof interest.

## Publisher's Note

All claims expressed in this article are solely those of the authors and do not necessarily represent those of their affiliated organizations, or those of the publisher, the editors and the reviewers. Any product that may be evaluated in this article, or claim that may be made by its manufacturer, is not guaranteed or endorsed by the publisher.
