# Supplementary material for: Development and Validation of a Two-Step Predictive Risk Stratification Model for Coronavirus Disease 2019 In-hospital Mortality: A Multicenter Retrospective Cohort Study
Source: Front Med (Lausanne). 2022 Apr 7;9:827261. doi: 10.3389/fmed.2022.827261 (PMC9021426; doi:10.3389/fmed.2022.827261)

**Supplementary Material**

**Contents**

**Supplementary Tables and Figures**

**Part I**

[Table S1: Demographic, Clinical, and Laboratory Characteristics of Derivation and Validation COVID-19 Cohorts 2](#_Toc96212328)

[Table S2: Variable Missing Details of VCU. 6](#_Toc96212329)

[Table S3: Variable Selection Count of Step 1. 7](#_Toc96212330)

[Table S4: Parameter Estimation and Odds Ratio of Step 1. 8](#_Toc96212331)

[Table S5: Variable Selection Count of Step 2. 9](#_Toc96212332)

[Table S6: Parameter Estimation and Odds Ratio of Step 2. 10](#_Toc96212333)

**Part II Sensitivity Analysis**

[Table S7: Parameter Estimation and Odds Ratio of CC-TS Step1. 11](#_Toc96212334)

[Table S8: Parameter Estimation and Odds Ratio of CC-TS Step2. 12](#_Toc96212335)

[Table S9: Discrimination Performance from CC-TS. 13](#_Toc96212336)

[Figure S1: Calibration Plots of the CC-TS. 14](#_Toc96212337)

**Part III Comparison With the Direct Method**

[Table S10: Variable Selection Count of Direct One-step Method. 15](#_Toc96212338)

[Table S11: Parameter Estimation and Odds Ratio of Direct One-step Method. 16](#_Toc96212339)

[Table S12: Risk Stratification among Four Cohorts of Direct One-step Method. 17](#_Toc96212340)

[Table S13: Extra Patients Triaged by the Two-step Method vs. the Direct One-step Method. 18](#_Toc96212341)

[Figure S2: Calibration Plot of the One-step Method. 19](#_Toc96212343)

Table S1: Demographic, Clinical, and Laboratory Characteristics of Derivation and Validation COVID-19 Cohorts

| **Characteristic** | **VCU**  **(N = 1673)** | **GU**  **(N = 558)** | **VFL**  **(N = 1815)** | **UCLA**  **(N = 1570)** |
| --- | --- | --- | --- | --- |
| **Death Count** | 180 (11%) | 93 (17%) | 269 (15%) | 184 (12%) |
| P-value |  | <0.001 | <0.001 | 0.418 |
| **Age** | 59 (45, 69) | 61 (50, 72) | 59 (44, 69) | 59 (44, 72) |
| P-value |  | <0.001 | 0.835 | 0.012 |
| Age group |  |  |  |  |
| <55 | 670 (40%) | 191 (34%) | 741 (41%) | 631 (40%) |
| 55-65 | 394 (24%) | 135 (24%) | 403 (22%) | 335 (21%) |
| 65-75 | 361 (22%) | 127 (23%) | 373 (21%) | 274 (17%) |
| ≥75 | 246 (15%) | 105 (19%) | 298 (16%) | 330 (21%) |
| P-value |  | 0.037 | 0.408 | <0.001 |
| **Gender** |  |  |  |  |
| Female | 820 (49%) | 284 (51%) | 929 (51%) | 706 (45%) |
| Male | 853 (51%) | 274 (49%) | 886 (49%) | 864 (55%) |
| P-value |  | 0.471 | 0.212 | 0.023 |
| **Heart Rate, per min** | 86 (75, 98) | 90 (78, 98) | 95 (82, 109) | 100 (88, 114) |
| P-value |  | 0.066 | <0.001 | <0.001 |
| Heart Rate Group |  |  |  |  |
| ≤100 | 1,273 (79%) | 469 (85%) | 1,116 (62%) | 558 (51%) |
| >100 | 341 (21%) | 82 (15%) | 694 (38%) | 545 (49%) |
| P-value |  | 0.002 | <0.001 | <0.001 |
| **Respiratory, per min** | 20.0 (17.0, 23.0) | 19.0 (18.0, 20.0) | 19.0 (16.0, 22.0) | 25.0 (21.0, 30.0) |
| P-value |  | 0.005 | 0.004 | <0.001 |
| Respiratory Group |  |  |  |  |
| <30 | 1,532 (92%) | 527 (96%) | 1,704 (94%) | 787 (71%) |
| ≥30 | 127 (7.7%) | 22 (4.0%) | 111 (6.1%) | 314 (29%) |
| P-value |  | 0.004 | 0.084 | <0.001 |
| **SpO_2_, %** | 96.0 (94.0, 98.0) | 96.0 (95.0, 98.0) | 95.0 (93.0, 98.0) | 97.5 (96.0, 99.0) |
| P-value |  | 0.238 | <0.001 | <0.001 |
| SpO_2_ Group |  |  |  |  |
| ≥93 | 1,479 (89%) | 521 (95%) | 1,398 (78%) | 1,064 (99%) |
| <93 | 182 (11%) | 30 (5.4%) | 402 (22%) | 12 (1.1%) |
| P-value |  | <0.001 | <0.001 | <0.001 |
| **COPD** |  |  |  |  |
| No | 1,265 (76%) | 481 (87%) | 1,343 (74%) | 1,184 (75%) |
| Yes | 408 (24%) | 74 (13%) | 472 (26%) | 386 (25%) |
| P-value |  | <0.001 | 0.289 | 0.928 |

Table S1: Continued

| **Characteristic** | **VCU**  **(N = 1673)** | **GU**  **(N = 558)** | **VFL**  **(N = 1815)** | **UCLA**  **(N = 1570)** |
| --- | --- | --- | --- | --- |
| **Asthma** |  |  |  |  |
| No | 1,352 (81%) | 492 (88%) | 1,489 (82%) | 1,418 (90%) |
| Yes | 321 (19%) | 66 (12%) | 326 (18%) | 152 (9.7%) |
| P-value |  | <0.001 | 0.375 | <0.001 |
| **CVD** |  |  |  |  |
| No | 1,025 (61%) | 469 (84%) | 1,259 (69%) | 1,058 (67%) |
| Yes | 648 (39%) | 89 (16%) | 556 (31%) | 512 (33%) |
| P-value |  | <0.001 | <0.001 | <0.001 |
| **Diabetes** |  |  |  |  |
| No | 802 (48%) | 327 (59%) | 922 (51%) | 1,033 (66%) |
| Yes | 871 (52%) | 231 (41%) | 893 (49%) | 537 (34%) |
| P-value |  | <0.001 | 0.098 | <0.001 |
| **Commodity Count** |  |  |  |  |
| ≤1 | 973 (58%) | 444 (80%) | 1,187 (65%) | 1,104 (70%) |
| ≥2 | 700 (42%) | 111 (20%) | 628 (35%) | 466 (30%) |
| P-value |  | <0.001 | <0.001 | <0.001 |
| **BMI** | 31 (25, 37) | 30 (26, 37) | 31 (26, 37) | 29 (25, 34) |
| P-value |  | 0.445 | 0.413 | <0.001 |
| BMI Group |  |  |  |  |
| 18.5-25 | 303 (21%) | 104 (19%) | 347 (20%) | 171 (24%) |
| <18.5 | 43 (2.9%) | 3 (0.5%) | 28 (1.7%) | 14 (1.9%) |
| 25-30 | 346 (23%) | 160 (29%) | 404 (24%) | 210 (29%) |
| ≥30 | 781 (53%) | 279 (51%) | 914 (54%) | 325 (45%) |
| P-value |  | 0.001 | 0.121 | 0.001 |
| **Temperature, ℃** | 36.90 (36.70, 37.20) | 37.20 (36.80, 37.60) | 37.11 (36.78, 37.78) | 37.44 (37.00, 38.22) |
| P-value |  | <0.001 | <0.001 | <0.001 |
| Fever |  |  |  |  |
| No | 1,245 (76%) | 246 (45%) | 982 (54%) | 414 (38%) |
| Yes | 390 (24%) | 302 (55%) | 830 (46%) | 670 (62%) |
| P-value |  | <0.001 | <0.001 | <0.001 |
| **NEU, × 10⁹ / L** | 4.8 (3.0, 7.3) | 4.4 (3.1, 6.1) | 4.9 (3.4, 7.5) | 5.3 (3.5, 8.1) |
| P-value |  | 0.007 | 0.306 | 0.277 |
| NEU Group |  |  |  |  |
| ≤6.3 | 413 (68%) | 418 (76%) | 1,148 (66%) | 476 (63%) |
| >6.3 | 196 (32%) | 129 (24%) | 583 (34%) | 283 (37%) |
| P-value |  | 0.001 | 0.532 | 0.056 |
| **LYM, × 10⁹ / L** | 0.90 (0.60, 1.30) | 1.10 (0.80, 1.50) | 1.01 (0.65, 1.46) | 0.96 (0.65, 1.36) |
| P-value |  | 0.788 | 0.547 | 0.016 |

Table S1: Continued

| **Characteristic** | **VCU**  **(N = 1673)** | **GU**  **(N = 558)** | **VFL**  **(N = 1815)** | **UCLA**  **(N = 1570)** |
| --- | --- | --- | --- | --- |
| LYM Group |  |  |  |  |
| ≥1.1 | 232 (38%) | 298 (54%) | 759 (44%) | 302 (40%) |
| <1.1 | 377 (62%) | 249 (46%) | 971 (56%) | 456 (60%) |
| P-value |  | <0.001 | 0.015 | 0.547 |
| **NLR** | 5.5 (3.0, 9.6) | 4.0 (2.5, 6.4) | 4.8 (2.9, 8.8) | 5.6 (3.2, 9.8) |
| P-value |  | <0.001 | 0.219 | 0.787 |
| NLR Group |  |  |  |  |
| ≤3.7 | 395 (65%) | 469 (86%) | 1,199 (70%) | 513 (68%) |
| >3.7 | 214 (35%) | 78 (14%) | 525 (30%) | 244 (32%) |
| P-value |  | <0.001 | 0.037 | 0.283 |
| **WBC, × 10⁹/L** | 6.6 (4.6, 9.6) | 6.3 (4.8, 8.0) | 6.8 (5.1, 9.5) | 7.3 (5.3, 10.2) |
| P-value |  | 0.001 | 0.777 | 0.021 |
| WBC Group |  |  |  |  |
| ≤9.5 | 1,001 (74%) | 461 (83%) | 1,343 (75%) | 780 (71%) |
| >9.5 | 346 (26%) | 92 (17%) | 449 (25%) | 324 (29%) |
| P-value |  | <0.001 | 0.718 | 0.048 |
| **Creatinine, mg/dL** | 0.98 (0.75, 1.48) | 1.03 (0.79, 1.44) | 1.02 (0.78, 1.45) | 0.91 (0.71, 1.27) |
| P-value |  | 0.386 | 0.570 | 0.028 |
| Creatinine Group |  |  |  |  |
| 0.6-1.3 | 823 (59%) | 357 (65%) | 1130 (64%) | 697 (64%) |
| <0.6 | 136 (9.8%) | 38 (6.9%) | 109 (6.2%) | 137 (13%) |
| >1.3 | 429 (31%) | 158(29%) | 520 (30%) | 256 (24%) |
| P-value |  | 0.051 | <0.001 | 0.034 |
| **Platelets, × 10⁹ / L** | 204 (153, 265) | 203 (158, 259) | 213 (163, 272) | 214 (164, 278) |
| P-value |  | 0.837 | 0.005 | 0.006 |
| Platelet Group |  |  |  |  |
| >350 | 110 (8.2%) | 41 (7.5%) | 174 (9.8%) | 112 (10%) |
| ≤350 | 1,230 (92%) | 503 (92%) | 1,606 (90%) | 991 (90%) |
| P-value |  | 0.694 | 0.149 | 0.111 |
| **BUN, mg/dL** | 17 (12, 29) | 16 (11, 26) | 16 (11, 26) | 15 (11, 24) |
| P-value |  | 0.096 | 0.001 | <0.001 |
| BUN Group |  |  |  |  |
| ≤20 | 837 (61%) | 363 (66%) | 1,151 (65%) | 664 (67%) |
| >20 | 542 (39%) | 190 (34%) | 608 (35%) | 327 (33%) |
| P-value |  | 0.048 | 0.007 | 0.002 |
| **AST, U/L** | 40 (27, 65) | 40 (27, 63) | 32 (22, 48) | 39 (27, 58) |
| P-value |  | 0.819 | 0.027 | 0.302 |

Table S1: Continued

| **Characteristic** | | **VCU**  **(N = 1673)** | | **GU**  **(N = 558)** | | **VFL**  **(N = 1815)** | | **UCLA**  **(N = 1570)** | |
| --- | --- | --- | --- | --- | --- | --- | --- | --- | --- |
| AST Group | |  | |  | |  | |  | |
| ≤40 | | 516 (51%) | | 231 (51%) | | 993 (65%) | | 471 (53%) | |
| >40 | | 498 (49%) | | 222 (49%) | | 531 (35%) | | 416 (47%) | |
| P-value | |  | | 1.000 | | <0.001 | | 0.369 | |
| **CRP, mg/L** | | 6.7 (2.6, 13.6) | | 8.5 (3.8, 13.1) | | 7.1 (2.7, 13.8) | | 8.1 (3.3, 13.4) | |
| P-value | |  | | 0.919 | | 0.207 | | 0.512 | |
| CRP Group |  | |  | |  | |  | |  |
| ≤10 | | 467 (65%) | | 184 (61%) | | 835 (62%) | | 408 (60%) | |
| >10 | | 246 (35%) | | 119 (39%) | | 516 (38%) | | 268 (40%) | |
| P-value | |  | | 0.168 | | 0.109 | | 0.054 | |
| **Troponin-I, ng/ml** | | 0.04 (0.02, 0.20) | | 0.02 (0.02, 0.03) | | 0.06 (0.03, 0.17) | | 0.04 (0.04, 0.04) | |
| P-value | |  | | 0.616 | | 0.035 | | 0.092 | |
| Troponin-I Group | |  | |  | |  | |  | |
| ≤0.04 | | 134 (51%) | | 338 (77%) | | 33 (37%) | | 686 (85%) | |
| >0.04 | | 127 (49%) | | 99 (23%) | | 57 (63%) | | 125 (15%) | |
| P-value | |  | | <0.001 | | 0.023 | | <0.001 | |

Statistics presented: Median (IQR); n (%)

P-values were derived from comparison tests between the derivation cohort and each validation cohort. Pearson χ2 tests or unpaired T tests were employed as appropriate.

Abbreviations：

SpO_2_: Peripheral Capillary Oxygen Saturation CVD: Cardiovascular disease

COPD: Chronic Obstructive Pulmonary Disease BMI: Body mass index

NEU: Neutrophils LYM: Lymphocytes

WBC: White Blood Cell Count BUN: Blood Urea Nitrogen

AST: Aspartate Aminotransferase CRP: C-Reactive Protein

*Fever: Yes represents temperature>37.2℃, No means temperature≤37.2℃.

Table S2: Variable Missing Details of VCU.

| **mis_name** | **mis_sum** | **mis_prop** |
| --- | --- | --- |
| TroponinI | 1412 | 0.844 |
| Lymphs | 1064 | 0.636 |
| Neutro | 1064 | 0.636 |
| NLR | 1064 | 0.636 |
| CRP | 960 | 0.574 |
| AST | 659 | 0.394 |
| Platelets | 333 | 0.199 |
| WBC | 326 | 0.195 |
| BUN | 294 | 0.176 |
| Creatinine | 285 | 0.17 |
| BMI | 200 | 0.12 |
| Heart rate | 59 | 0.035 |
| Temperature | 38 | 0.023 |
| Resprate | 14 | 0.008 |
| SpO2 | 12 | 0.007 |
| Age | 2 | 0.001 |

Miss_name: names of missing variables in GU cohort;

Miss_count: missing count for each missing variable;

Miss_prop: missing proportion of each missing variable.

Table S3: Variable Selection Count of Step 1.

| **Variable** | **Frequency** |
| --- | --- |
| Age | 100 |
| Respiratory rate | 100 |
| SpO_2_ | 100 |
| Commodity count | 100 |
| Heart rate | 100 |
| BMI | 5 |

* All the variables are categorical.

Table S4: Parameter Estimation and Odds Ratio of Step 1.

|  | **Estimate** | **P value** | **Odds Ratio** | **95%CI** |
| --- | --- | --- | --- | --- |
| (Intercept) | -3.49 | <0.001 | 0.03 | (0.02,0.05) |
| Age 55-64 | 0.75 | 0.004 | 2.11 | (1.27,3.51) |
| Age 65-74 | 1.26 | <0.001 | 3.52 | (2.16,5.72) |
| Age ≥75 | 1.76 | <0.001 | 5.79 | (3.53,9.51) |
| Commodity count≥2 | 0.45 | 0.008 | 1.57 | (1.13,2.19) |
| Heart rate>100 | 0.37 | 0.068 | 1.45 | (0.97,2.15) |
| Respiratory rate ≥ 30 | 0.81 | 0.001 | 2.24 | (1.38,3.65) |
| SpO_2_ < 93% | 0.65 | 0.003 | 1.92 | (1.26,2.94) |

Table S5: Variable Selection Count of Step 2.

| **Variable** | **Frequency** |
| --- | --- |
| BUN | 100 |
| Age | 100 |
| SpO2 | 100 |
| NEU | 88 |
| NLR | 75 |
| Platelets | 73 |
| CRP | 69 |
| WBC | 11 |
| Respiratory rate | 3 |
| Lym | 1 |

*All the variables are categorical.

Table S6: Parameter Estimation and Odds Ratio of Step 2.

|  | **Estimate** | **P value** | **Odds Ratio** | **95%CI** |
| --- | --- | --- | --- | --- |
| (Intercept) | -5.36 | <0.001 | 0.00 | (0.00,0.02) |
| BUN>20 | 1.18 | <0.001 | 3.25 | (3.25,4.97) |
| Age 55-64 | 0.47 | 0.130 | 1.61 | (1.61,2.96) |
| Age 65-74 | 0.74 | 0.019 | 2.09 | (2.09,3.85) |
| Age ≥75 | 1.21 | 0.001 | 3.36 | (3.36,6.58) |
| SpO_2_ <93% | 0.85 | 0.004 | 2.35 | (2.35,4.19) |
| NEU >6.3 | 0.89 | 0.001 | 2.43 | (2.43,3.98) |
| NLR >7.9 | 0.98 | 0.023 | 2.66 | (2.66,5.45) |
| CRP >10 | 0.54 | 0.115 | 1.71 | (1.71,3.17) |
| Platelets ≥350 | 1.02 | 0.031 | 2.77 | (2.77,6.93) |

**Part II: Sensitivity Analysis**

We conducted a sensitivity analysis using the two-step method on the complete cases with our missing data imputation, and named this approach CC-TS.

Table S7: Parameter Estimation and Odds Ratio of CC-TS Step1.

|  | Estimate | P value | aOR | 95%CI | Point |
| --- | --- | --- | --- | --- | --- |
| (Intercept) | -3.41 | <0.001 | 0.03 | (0.02,0.05) | 0 |
| Age 55-64 | 0.87 | 0.002 | 2.39 | (1.38,4.14) | 2 |
| Age 65-74 | 1.33 | <0.001 | 3.79 | (2.24,6.42) | 4 |
| Age ≥75 | 1.71 | <0.001 | 5.53 | (3.21,9.52) | 5 |
| Respiratory rate ≥ 30 | 0.89 | 0.001 | 2.42 | (1.46,4.03) | 2 |
| SpO_2_ <93% | 0.58 | 0.011 | 1.78 | (1.14,2.78) | 2 |
| Commodity count≥2 | 0.37 | 0.043 | 1.44 | (1.01,2.06) | 1 |

The ‘Point’ column showed score of each risk factor level.

Table S8: Parameter Estimation and Odds Ratio of CC-TS Step2.

|  | **Estimate** | **P value** | **Odds Ratio** | **95%CI** | **Point** |
| --- | --- | --- | --- | --- | --- |
| (Intercept) | -4.29 | <0.001 | 0.01 | (0.00,0.07) | 0 |
| BUN >20 | 1.53 | <0.001 | 4.62 | (2.06,10.37) | 2 |
| NEU >6.3 | 1.14 | 0.002 | 3.13 | (1.52,6.47) | 2 |
| CRP >10 | 0.76 | 0.039 | 2.13 | (1.04,4.39) | 1 |
| Platelets ≥350 | 1.15 | 0.103 | 3.17 | (0.79,12.72) | 2 |

The ‘Point’ column showed score of each risk factor level.

Table S9: Discrimination Performance from CC-TS.

|  | **AUC** | **95%CI** |
| --- | --- | --- |
| VCU | 0.81 | (0.76,0.86) |
| GU | 0.83 | (0.77,0.89) |
| UFL | 0.78 | (0.75,0.81) |
| UCLA | 0.80 | (0.75,0.85) |

Figure S1: Calibration Plots of the CC-TS.


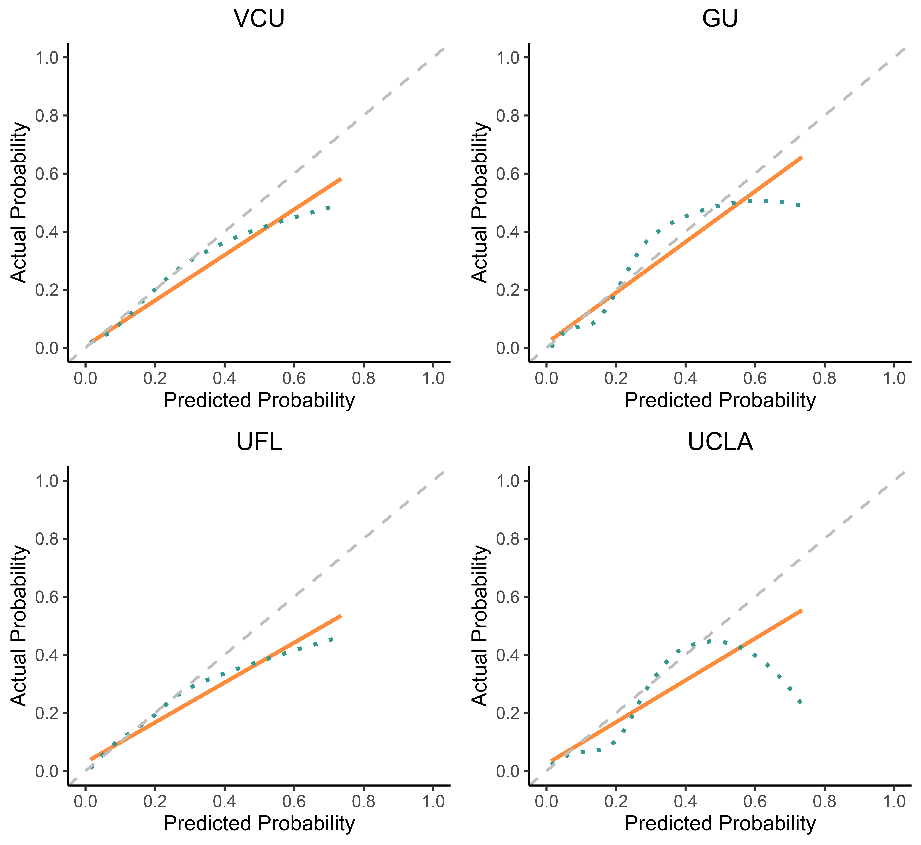


**Part III: Comparison With the Direct Method**

Table S10: Variable Selection Count of Direct One-step Method.

| **Variable** | **Frequency** |
| --- | --- |
| BUN | 100 |
| Age | 100 |
| NLR | 91 |
| CRP | 74 |
| Platelets | 73 |
| WBC | 67 |
| SpO2 | 65 |
| Respiratory rate | 48 |
| NEU | 32 |
| Commodity Count | 6 |

*All the variables are categorical.

Table S11: Parameter Estimation and Odds Ratio of Direct One-step Method.

|  | **Estimate** | **P Value** | **Odds Ratio** | **95%CI** |
| --- | --- | --- | --- | --- |
| (Intercept) | -5.37 | <0.001 | 0.00 | (0.00,0.01) |
| BUN >20 | 1.17 | <0.001 | 3.23 | (3.23,4.75) |
| Age 55-64 | 0.76 | 0.006 | 2.13 | (2.13,3.63) |
| Age 65-74 | 0.98 | <0.001 | 2.65 | (2.65,4.48) |
| Age ≥75 | 1.25 | <0.001 | 3.48 | (3.48,5.99) |
| Platelets ≥350 | 0.85 | 0.030 | 2.34 | (2.34,5.02) |
| CRP >10 | 0.76 | 0.012 | 2.15 | (2.15,3.60) |
| NLR >7.9 | 0.95 | 0.008 | 2.59 | (2.59,4.66) |
| WBC >9.5 | 0.76 | 0.002 | 2.14 | (2.14,3.36) |
| SpO_2_ <93% | 0.52 | 0.037 | 1.68 | (1.68,2.73) |

Table S12: Risk Stratification among Four Cohorts of Direct One-step Method.

|  | **Internal validation** | **External validation cohorts** | | | |
| --- | --- | --- | --- | --- | --- |
| **Risk group** | **VCU** | **GU** | **UFL** | **UCLA** | **All validation cohorts** |
| **Lower risk** | 0.0%  (0/52) | 1.6%  (1/62) | 1.4%  (3/218) | 0.0%  (0/87) | 1.1%  (4/367) |
| **Moderate risk** | 4.3%  (6/141) | 6.9%  (10/144) | 6.5%  (35/536) | 9.3%  (17/183) | 7.2%  (62/863) |
| **Higher risk** | 32.9%  (51/155) | 38.6%  (34/88) | 31.2%  (177/568) | 27.3%  (51/187) | 31.1%  (262/843) |
| **AUROCC:** | 0.823 | 0.806 | 0.793 | 0.794 | 0.798 |

Table S13: Extra Patients Triaged by the Two-step Method vs. the Direct One-step Method.

|  | **S1*** | **OS_in_S1#** | **Extra** |
| --- | --- | --- | --- |
| **VCU** | 390 | 59  67  262  87 | 331(84.87%) |
| **GU** | 144 |  | 77(53.47%) |
| **UFL** | 427 |  | 165(38.64%) |
| **UCLA** | 223 |  | 136(60.99%) |

*S1: the number of patients identified as lower risk by the first step of the two-step method (TS);

#OS_in_S1: the number of patients triaged in S1 who could also be identified by the direct one-step method (OS);

Extra: the number of patients in S1 who were uncategorized by OS for a lack of further laboratory tests.

Figure S2: Calibration Plot of the One-step Method.


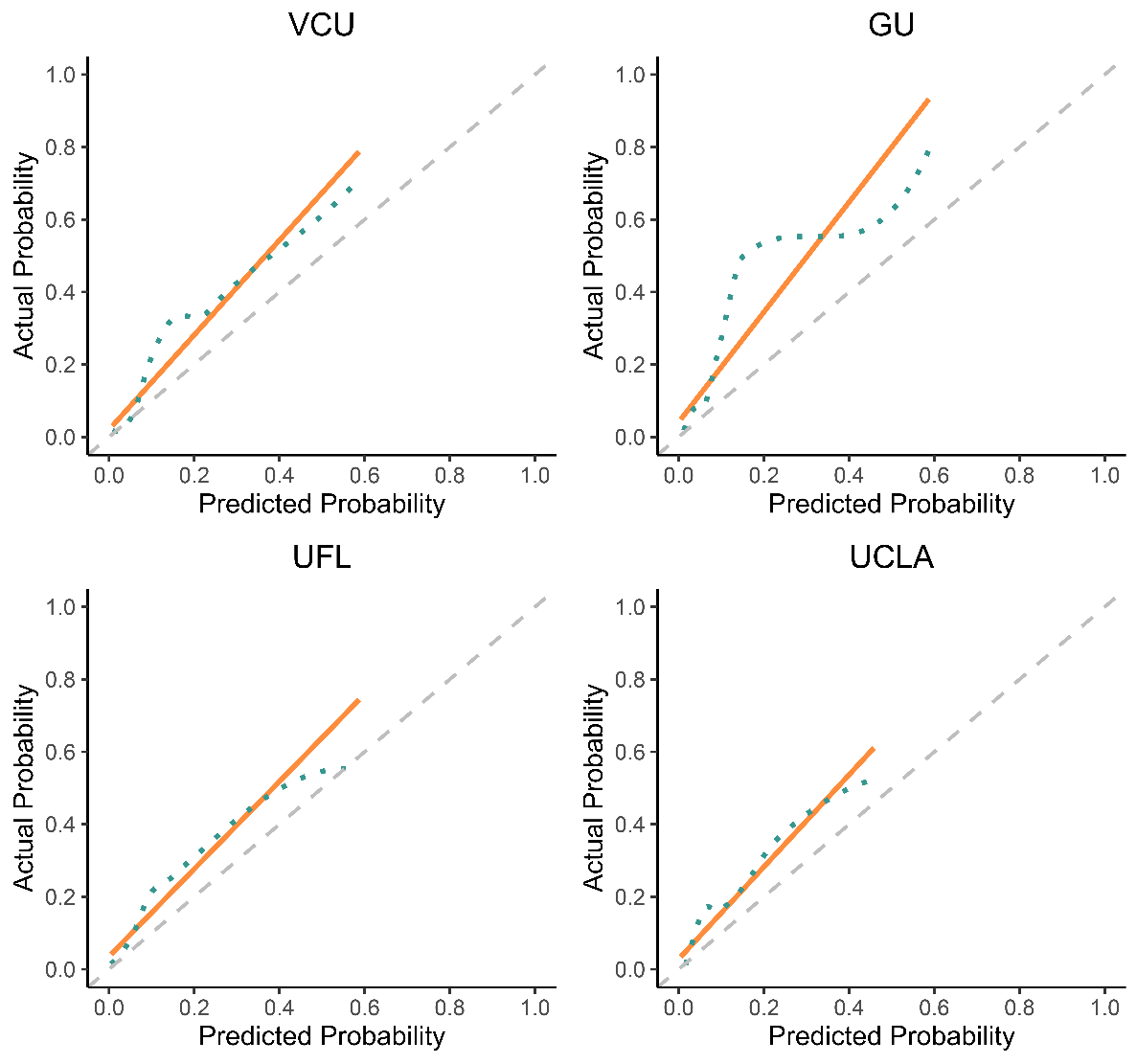

Supplement: Supplementary file 1 [file Data_Sheet_1.docx]
